# 17 A Tissue-engineered Bi-layer Skin Substitute Polarizes Macrophages Towards an M2 Phenotype

**DOI:** 10.1093/jbcr/irac012.021

**Published:** 2022-03-23

**Authors:** Beatriz Hernaez-Estrada, Lindsay Steele, Kara Spiller

**Affiliations:** Drexel University, Philadelphia, Pennsylvania; Drexel University, Philadelphia, Pennsylvania; Drexel University, Philadelphia, Pennsylvania

## Abstract

**Introduction:**

Cellular and/or Tissue Product made of stratified keratinocyte-containing epidermis and fibroblast-embedded collagen dermis, has been shown to promote healing in patients with deep partial- thickness burns. The biological mechanisms behind these effects are poorly understood. Macrophages, the primary cell type of the innate immune system, are major regulators of all stages of tissue repair. They accomplish these diverse functions by shifting phenotype throughout the healing process from a pro- inflammatory population to a diverse population of phenotypes that orchestrate resolution of inflammation and healing. In burn injuries this transition is impaired. We hypothesized that skin substitutes promote this healthy transition in macrophage phenotype. The aim of this study was to analyze the crosstalk between skin substitutes and macrophages and to determine the influence of direct contact, secreted factors, and the substrate used in fabrication of the skin substitutes.

**Methods:**

Primary human macrophages (N=4 donors) were co-cultured either directly or indirectly with a skin substitute via separation by transwell inserts. Macrophages were also cultured directly on the collagen scaffolds used in the skin substitute fabrication process or on low-activation plastic as an unactivated control. After 1 and 3 days, the macrophages were stained with a 10-marker flow cytometry panel to characterize macrophage phenotype: general marker (CD45), M1-related markers (CCR7, CD80, CD38 and PD-L1) and M2-related markers (CD206, CD209, CD163 and CXCR4). For a more thorough evaluation, multidimensional analysis was used to identify unique populations of macrophages. Large-scale Dimensionality reduction using triplets and clustering analysis with using FlowSOM algorithms were performed. Statistical analysis of individual marker expression or cell composition within clusters was performed with one-way ANOVA.

**Results:**

In response to both direct and indirect interaction with skin substitutes, macrophages generally decreased M1 marker expression after 1 day in culture and increased M2 marker expression after 3 days. The suppression of M1 markers occurred when macrophages were co-cultured with skin substitutes, and in response to acellular collagen. The upregulation of M2 markers occurred only in response to direct or indirect interaction with cellular skin substitutes. Furthermore, multidimensional single cell analysis showed that contact with the skin substitutes also promoted unique populations of phenotypes that do not fit into the classical M1/M2 paradigm.

**Conclusions:**

These results suggest that a potential mechanism by which skin substitutes promote healing is by promoting a transition of macrophages towards pro-reparative phenotypes.

